# Supplementary material for: Temporal analysis of T-cell receptor-imposed forces via quantitative single molecule FRET measurements
Source: Nat Commun. 2021 May 4;12:2502. doi: 10.1038/s41467-021-22775-z (PMC8096839; doi:10.1038/s41467-021-22775-z)
Supplement: Supplementary file 1 — Supplementary Information [file 41467_2021_22775_MOESM1_ESM.pdf]

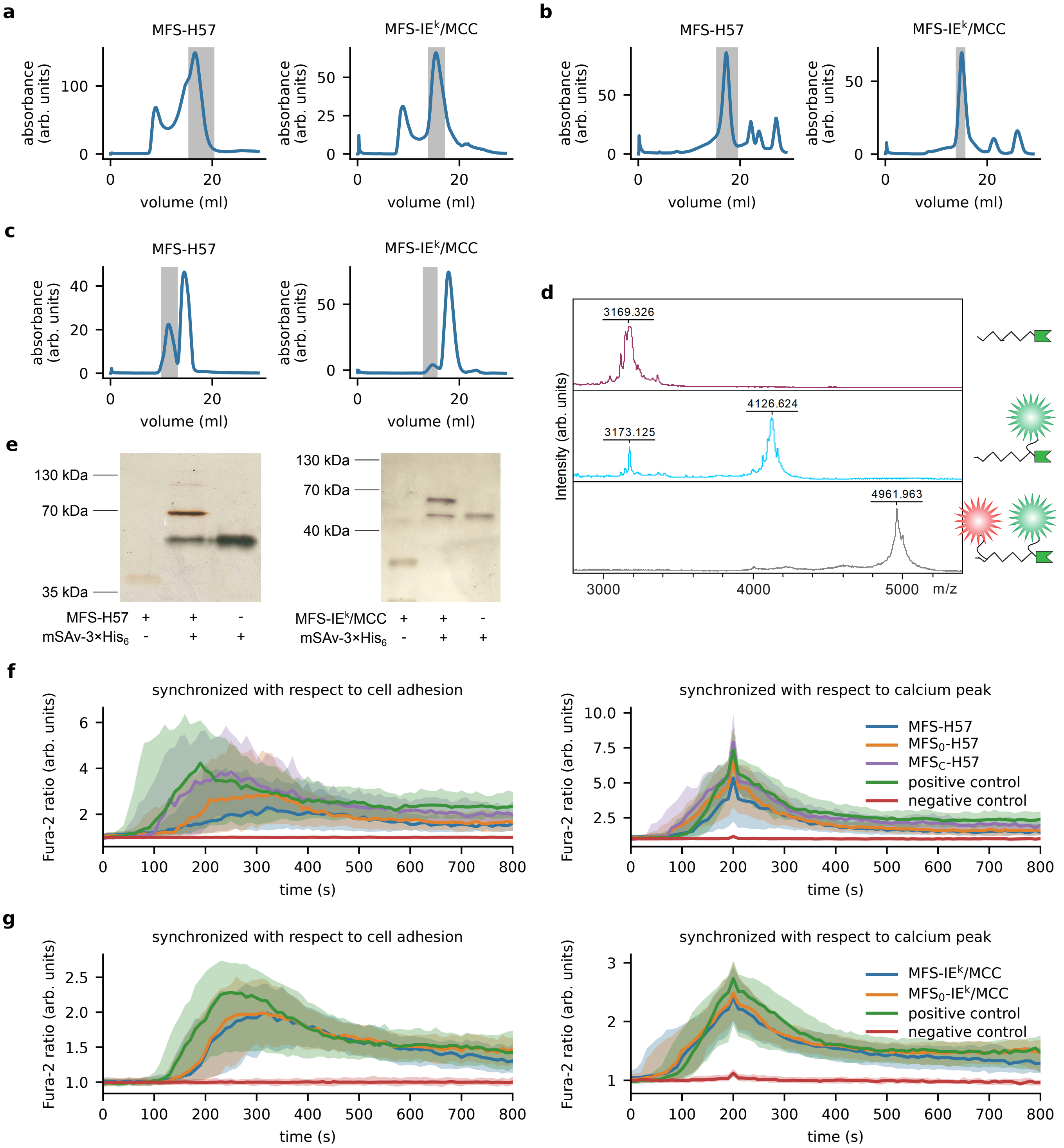

Supplementary Figure 1: *Synthesis and functional validation of the TCR-specific force sensor.* (a) Refolded single chain antibody fragment derived from the TCRβ-reactive H57 monoclonal antibody (H57-scF$_V$) and refolded murine pMHC class II molecule IE^k with MCC peptide (IE^k/MCC) was purified by Superdex 200 10/300 GL (S200) size exclusion chromatography. The gray areas correspond to collected fractions. (b) Post conjugation, the surplus of DBCO was removed from monomeric H57-scF$_V$-DBCO and IE^k/MCC-DBCO via Superdex 75 10/300 GL (S75) gel filtration. (c) Unconjugated MFS was separated from MFS-H57 or MFS-IE^k/MCC via S75 or S200 gel filtration, respectively. (d) Mass spectra of HPLC purified unlabeled, Alexa Flour 555-labeled and Alexa 555 / Alexa 647 dual-labeled MFS (top to bottom) confirmed successful conjugation. The theoretical molar weight of the unconjugated peptide is 3172 Da. (e) SDS-PAGE gel shift analysis of MFS-H57 and MFS-IE^k/MCC in the presence of mSAv–3×His$_6$ performed under non-boiling and non-reducing conditions testifies to the complete conjugation of the force sensor. In case of MFS-H57, a second slight band corresponding to a double shift and a third barely visible band corresponding to triple shift indicated the presence of a small fraction of H57-scF$_V$ conjugated to more than one sensor peptide. (f–g) 5c.c7 TCR-transgenic T-cell blasts were brought into contact with fluid-phase SLB decorated with 100 to 150 molecules µm^{-2} of force sensor bound to mSAv–3×His$_6$ together with His-tagged ICAM-1 and B7-1. Fura-2 emission was measured at 340 nm and 380 nm excitation. The left panels show the increase of intracellular calcium levels measured by ratiometric Fura-2 analysis for MFS-H57, MFS$_0$-H57, and MFS$_C$-H57 (f), and for MFS-IE^k/MCC and MFS$_0$-IE^k/MCC (g). The right panels show the average calcium traces after synchronization with respect to the single cell calcium peaks. SLBs lacking the force sensors served as negative control (only His-tagged ICAM-1), as positive control we used SLBs containing His-tagged ICAM-1, B7-1, and MCC-loaded IE^k. Median per time point normalized by median of negative control is shown; interquartile range is shown as shaded area; n cells per sample with $112 \leq n \leq 311$.

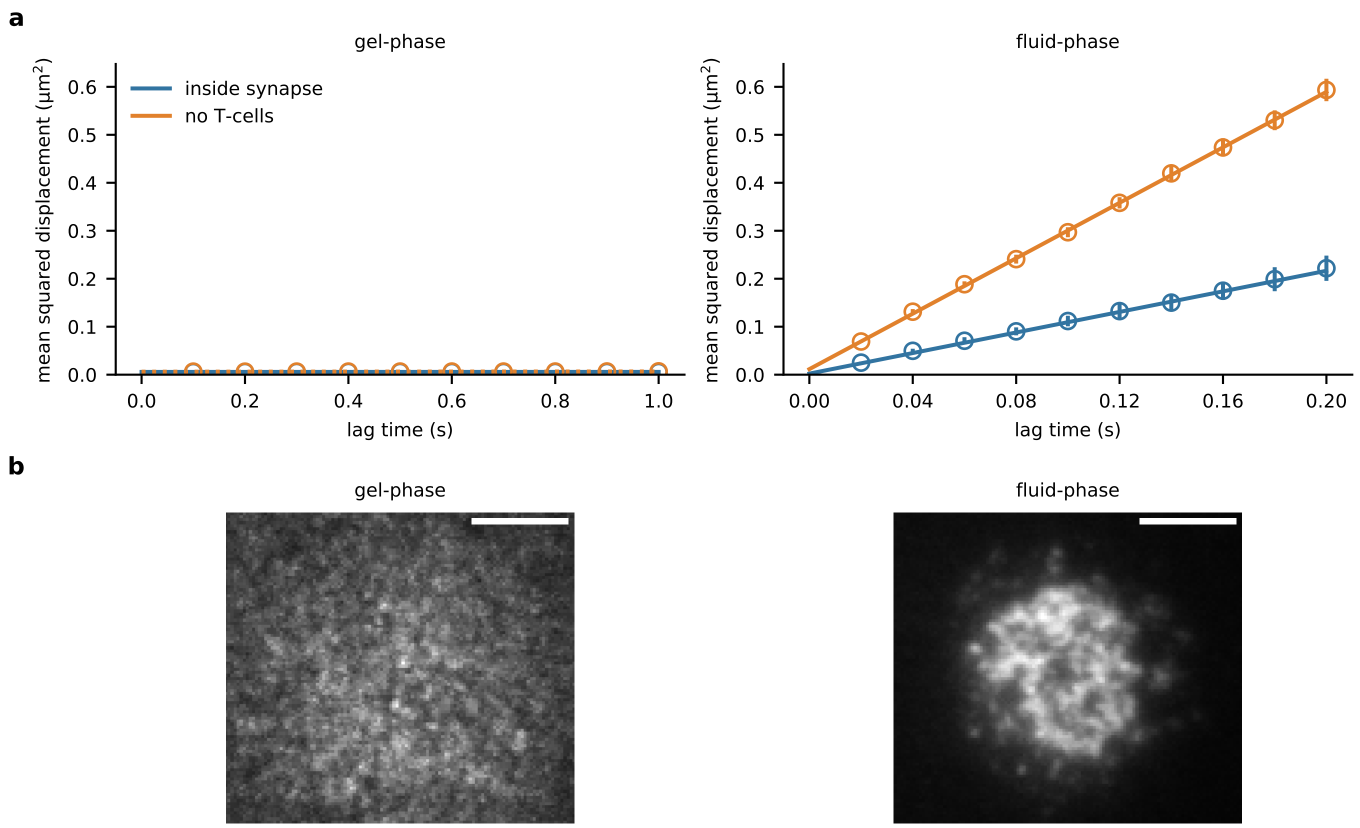

Supplementary Figure 2: *Diffusion analysis of the force sensor anchored to gel-phase and fluid phase SLBs and representative images of the immunological synapses.* (a) Diffusion analysis of MFS on DPPC membranes yielded $D = (5.8 \pm 8.6) \times 10^{-5}\,\mu m^2\,s^{-1}$ in the presence and $D = (13 \pm 6) \times 10^{-5}\,\mu m^2\,s^{-1}$ in the absence of T-cells (left panel). Analogous analysis employing POPC SLBs gave rise to $D = (0.27 \pm 0.02)\,\mu m^2\,s^{-1}$ in the presence and $D = (0.72 \pm 0.02)\,\mu m^2\,s^{-1}$ in the absence of T-cells (right panel). Reduced MFS mobility in the synapse arose from the presence of bound and immobilized molecules (compare Supplementary Figure 8). 2945 datapoints in 90 tracks from 77 movies for DPPC underneath cells, 3867 datapoints in 102 tracks from 30 movies for DPPC without cells, 724 datapoints in 29 tracks from 109 movies for POPC underneath cells, 904 datapoints in 31 tracks from 30 movies for POPC without cells. Error bars indicate standard errors. (b) TIRF imaging of MFS_C employed at high densities (see also Figure 1 c and d) showed microcluster formation within the immunological synapse of T-cells in contact with fluid-phase POPC-based SLBs (right panel) but not of T-cells engaging gel-phase DPPC-based SLBs (left panel). Scale bars $5\,\mu m$; $n = 27$ cells on gel-phase bilayers, $n = 21$ cells on fluid-phase bilayers.

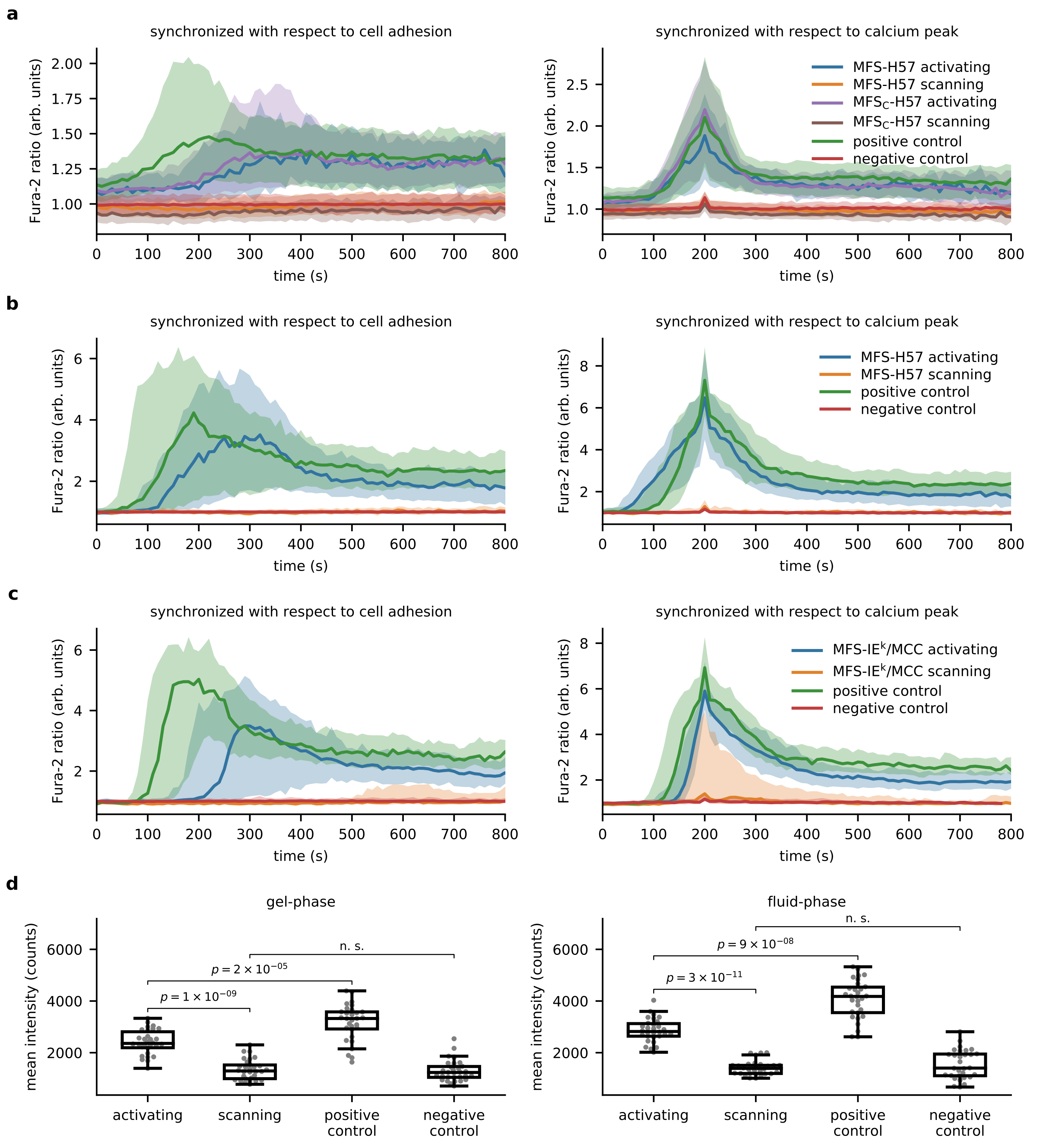

Supplementary Figure 3: *Functional response of T-cells in contact with gel-phase and fluid-phase SLBs displaying the force sensor.* (a–c) Ratiometric calcium imaging of T-cells confronted with gel-phase DPPC SLBs (a, c) and fluid-phase POPC SLBs (b). All SLBs employed featured His-tagged ICAM-1. In addition, one of the following (combination of) constructs were added: Panel (a): MFS-H57 (single-molecule density) + MFS$_0$-H57 (high density) + B7-1 (blue); MFS-H57 (single-molecule density) (orange); MFS$_C$-H57 (single-molecule density) + MFS$_0$-H57 (high density) + B7-1 (purple); MFS$_C$-H57 (single-molecule density) (brown). Panel (b): MFS-H57 (single-molecule density) + MFS$_0$-H57 (high density) + B7-1 (blue); MFS-H57 (single-molecule density) (orange). Panel (c): MFS-IE^k/MCC (single-molecule density) + MFS$_0$-IE^k/MCC (high density) + B7-1 (blue); MFS-IE^k/MCC (single-molecule density) (orange). In all panels, SLBs lacking the force sensors served as negative control (only His-tagged ICAM-1; red); as positive control we used SLBs containing His-tagged ICAM-1, B7-1, and MCC-loaded IE^k (green). Median per time point normalized by median of negative control is shown; interquartile range is shown as shaded area. The left panels show the average calcium traces after synchronization with respect to cell adhesion, the right panels after synchronization with respect to peak position. We used between 60 and 367 cells for analysis. (d) ZAP70 recruitment to the immunological synapse was assessed via immunostaining in conjunction with brightness analysis and TIRF microscopy. 5c.c7 T-cells were brought in contact with gel-phase (left panel) or fluid-phase SLBs (right panel), under activating or scanning conditions. T-cells were stimulated via MFS-H57. As positive control we used SLBs functionalized with ICAM-1, B7-1, and MCC-loaded IE^k, as negative control SLBs functionalized only with ICAM-1. Boxes indicate the interquartile range (first quartile to third quartile), whiskers extend 1.5 times the interquartile range from the first and third quartile, the central line indicates the median. $n = 30$ cells were recorded for each condition. p-values were calculated using the two-sample Mann–Whitney U test.

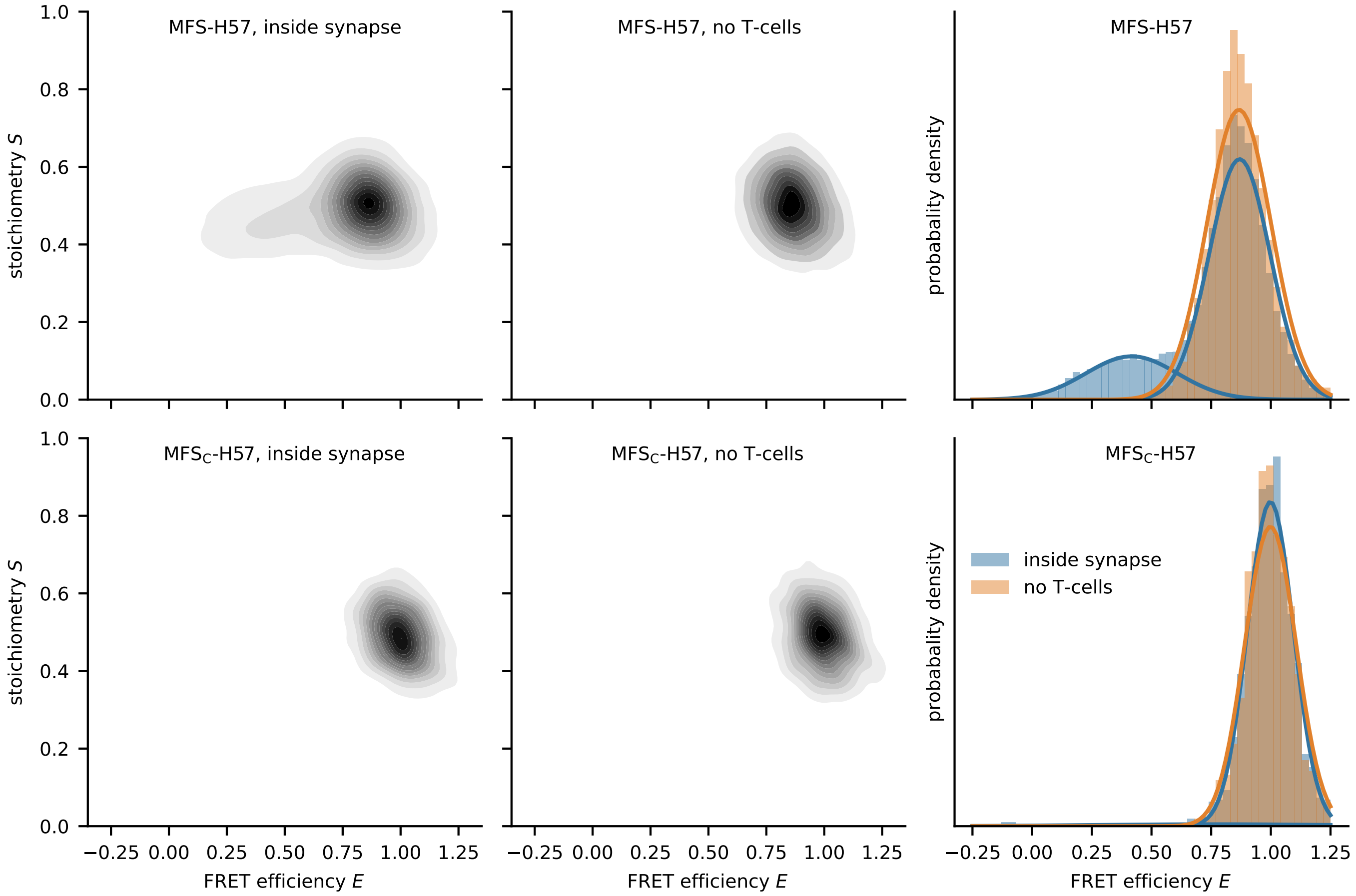

Supplementary Figure 4: *Reading out the force sensor at the single-molecule level.* FRET efficiency – stoichiometry (E–S) analysis of single-molecule datasets obtained for MFS and MFS$_C$ present on gel-phase SLBs in the absence and presence of T-cells. Each data point in the depicted cloud corresponds to one single-molecule FRET event. Measured FRET efficiencies E are indicated in histograms (right panels). A Gaussian mixture model was fitted to the E–S data, yielding pronounced maxima at $E = 0.87$ and $E = 0.41$ (MFS in the presence of T-cells), $E = 0.87$ (MFS in the absence of T-cells), $E = 0.99$ (MFS$_C$ in the presence of T-cells), and $E = 1.00$ (MFS$_C$ in the absence of T-cells). In the presence of T-cells the low FRET peak corresponds to a weight of 32 %. $n = 15\,556$ from 593 movies for MFS in the presence of T-cells, $n = 12\,657$ from 195 movies for MFS in the absence of T-cells, $n = 1451$ from 127 movies for MFS$_C$ in the presence of T-cells, $n = 1506$ from 30 movies for MFS$_C$ in the absence of T-cells.

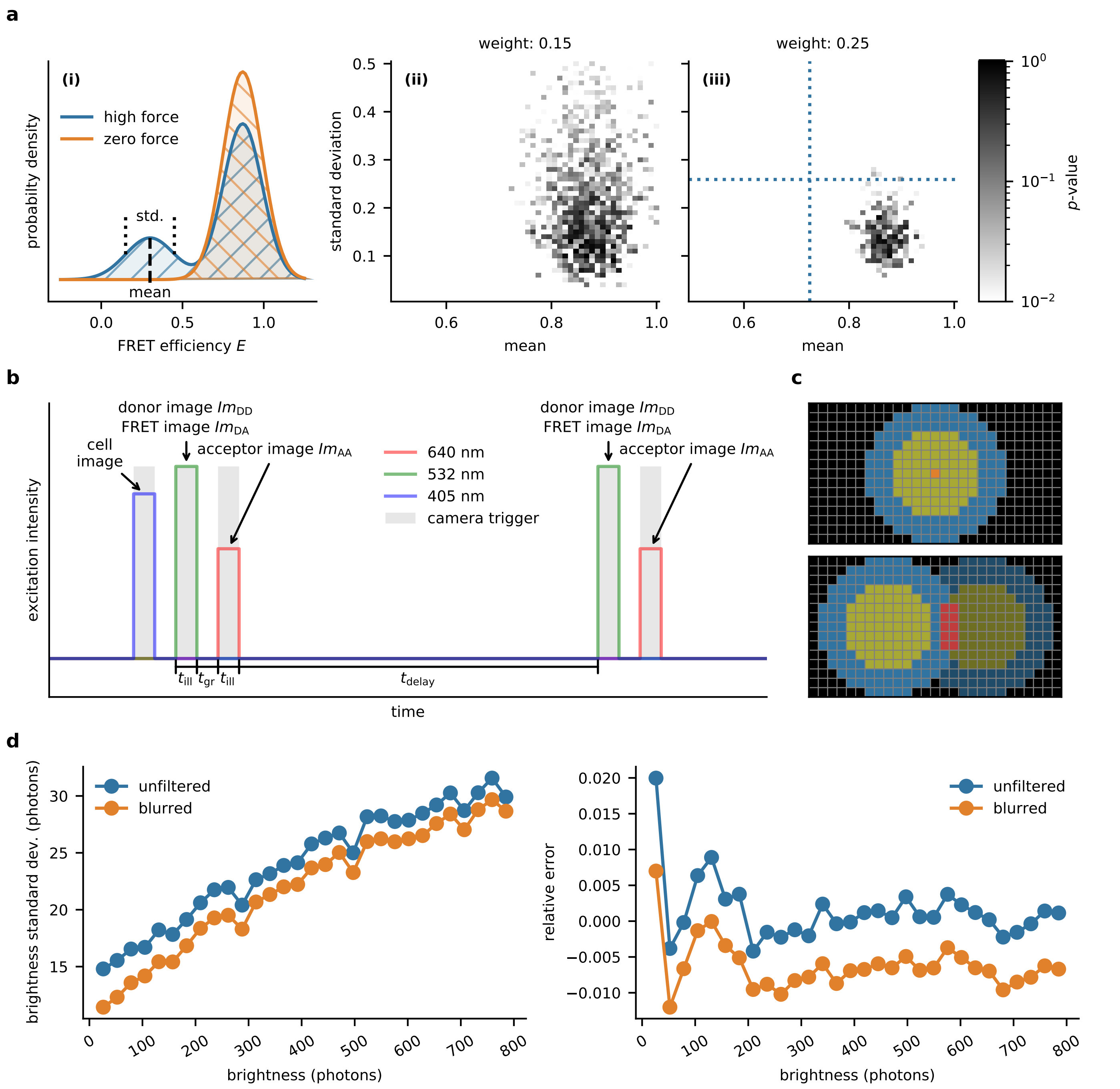

Supplementary Figure 5: *Quantitation of the single-molecule FRET signal.* (a) Determination of the lower boundary, F_{min}, of the sensitivity towards applied forces conveyed by the MFS. We simulated Gaussian-distributed random data sets for the high-FRET peak, taking a mean FRET efficiency of 0.87 and a standard deviation $\sigma = 0.12$. For the low FRET peak, we simulated different values for the mean FRET efficiency and the standard deviation (i). Samples were compared to a distribution lacking the low FRET peak using a two-sample Kolmogorov-Smirnov test. Determined p-values were plotted for the different parameter settings. Panel (ii) shows results assuming a statistical weight of 0.15 for the low FRET peak, panel (iii) for a statistical weight of 0.22. Dashed lines indicate the results from the experiments shown in Supplementary Figure 7 for MFS-H57 recorded on fluid phase SLBs under scanning conditions on 5c.c7 T cells (mean = 0.73, std = 0.26). (b) Illumination timing. Each image sequence is a repetition of two consecutive images recorded with 532 nm and 640 nm excitation, yielding Im_{DD}, Im_{DA}, and Im_{AA}. The timings are indicated in the image. Prior to this, samples were illuminated with 405 nm to obtain one image of the outline of cell in the Fura-2 channel. (c) Mask used for brightness analysis. If a molecule is located in the orange position, we calculated its brightness by summing over the yellow pixels, and subtracted the weighted blue pixels as background (top). If background of one signal overlaps with the foreground of another signal (bottom image), the corresponding affected pixels (red) were omitted in the analysis. (d) Gaussian blur reduces noise in brightness determination (left). For dim signals, noise is decreased by 25 % to 30 %. The blur leads to slight underestimation of the brightness (typically $<1\,\%$, right). Since this is constant over the brightness range, derived ratiometric quantities like E and S are not biased.

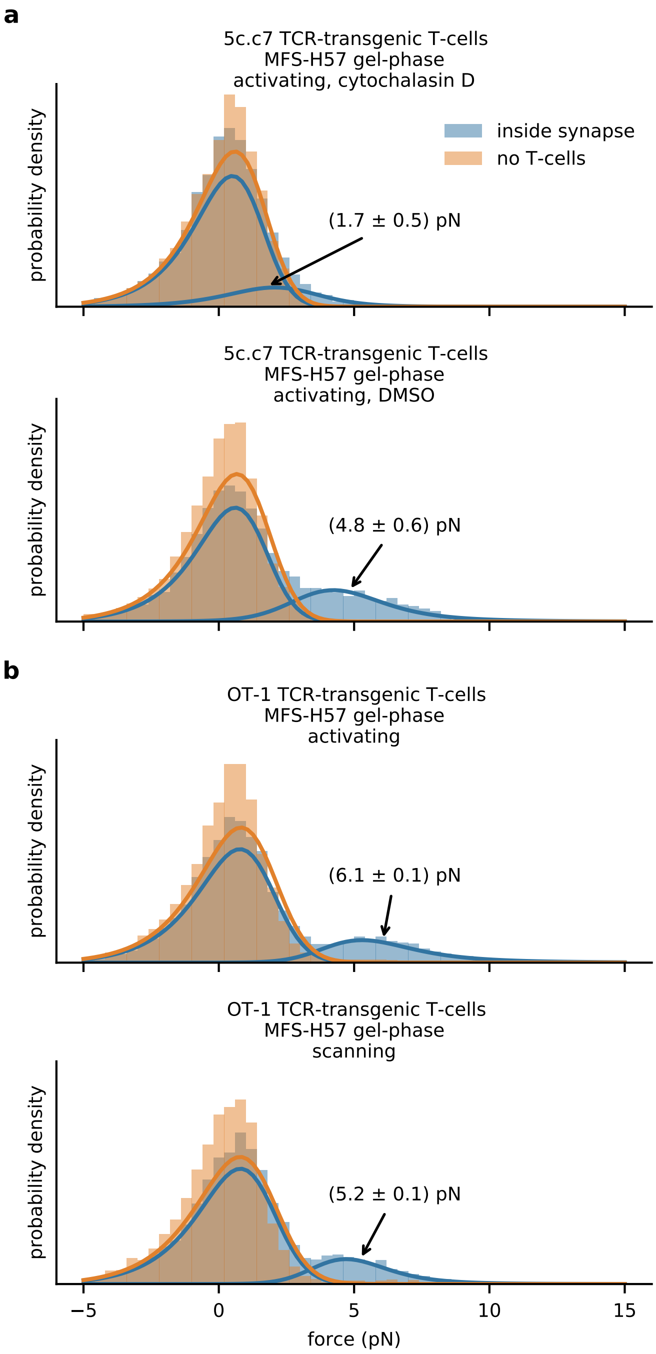

Supplementary Figure 6: *Influence of actin cytoskeleton and T-cell type on TCR-imposed forces.* (a) Single-molecule analysis of MFS-H57 on gel-phase SLBs recorded under activating conditions using 5c.c7 T-cells, upon inhibition of actin polymerization via cytochalasin D (top). The bottom panel shows the vehicle control. We observed a substantial reduction of the high force peak. $n = 5330$ (239 movies) for cytochalasin D-treated T-cells, $n = 5390$ (70 movies) without T-cells, $n = 4865$ (255 movies) for the vehicle control with T-cells, $n = 4637$ (69 movies) without T-cells. (b) Single-molecule analysis of MFS-H57 on gel-phase SLBs recorded under activating (top) or scanning conditions (bottom), using OT-1 $CD8^+$ T-cells. We observed a significant high force peak at $(6.1 \pm 0.1)\,\mathrm{pN}$ $(18\,\%)$ for the activating case and $(5.2 \pm 0.1)\,\mathrm{pN}$ $(15\,\%)$ under scanning conditions. $n = 8963$ (408 movies) for activated T-cells, $n = 5450$ (119 movies) for stimulatory SLBs without T-cells, $n = 5435$ (226 movies) for scanning T-cells, $n = 4572$ (66 movies) for non-stimulatory SLBs without T-cells.

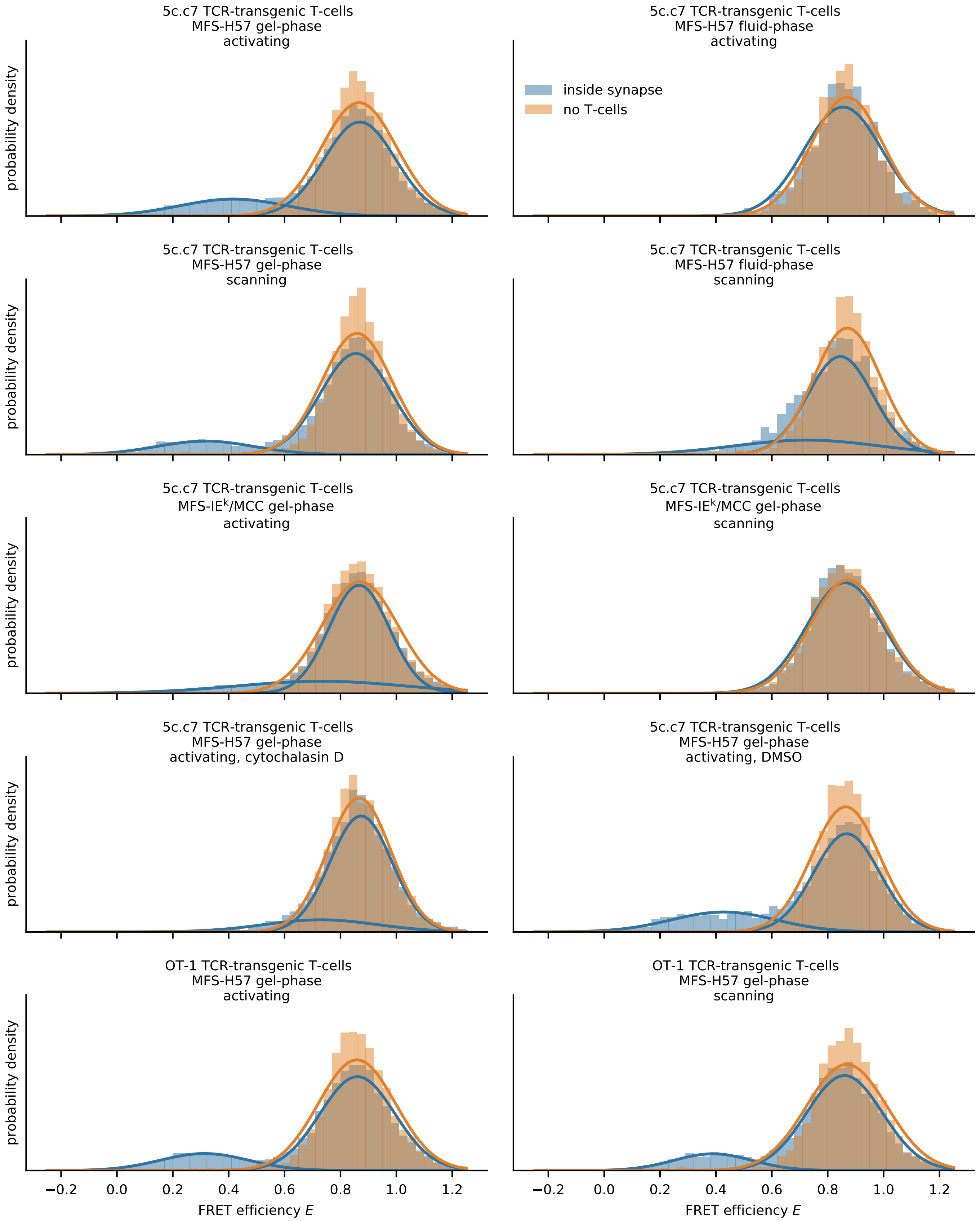

Supplementary Figure 7: *Histograms of single-molecule FRET data.* Histograms of FRET efficiencies E for the data sets shown in Figure 2, Figure 5, and Supplementary Figure 6. Lines indicate the fit results of the Gaussian mixture model for SLBs in contact with T-cells, and single Gaussian fits for SLBs without T-cells.

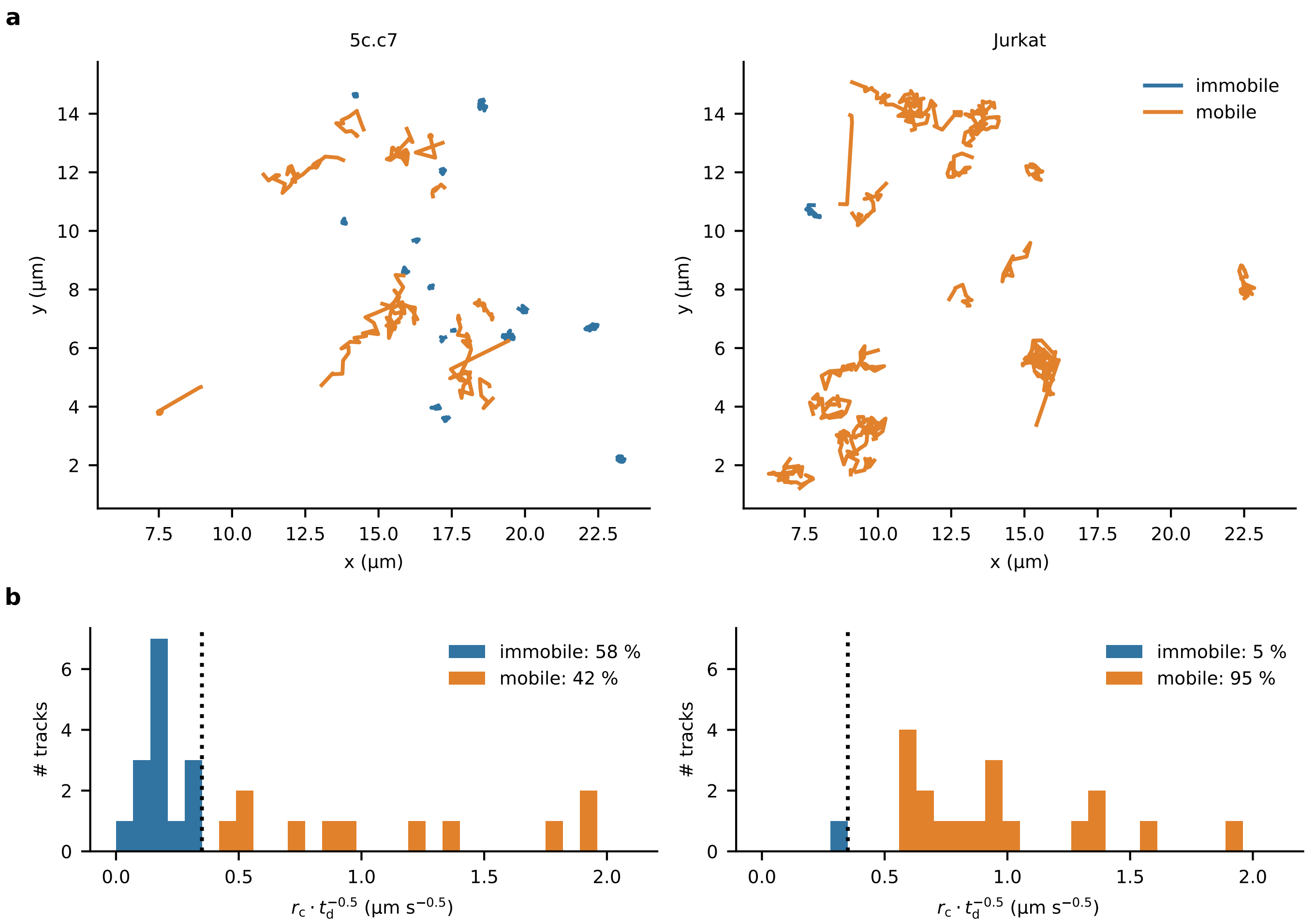

Supplementary Figure 8: *MFS mobility on POPC SLBs is reduced after synaptic TCR binding.* Mobility of MFS on POPC SLBs analyzed in a synapse involving 5c.c7 T-cells (left) or Jurkat T-cells serving as a negative control (right). Panel (a) shows selected trajectories, panel (b) a histogram of the calculated extension of the trajectory, r, normalized by the square root of its duration, t_d. The selection threshold is indicated as dashed line. Diffusion analysis of MFS yielded: $D = (0.018 \pm 0.007)\,\mu\mathrm{m}^2\,\mathrm{s}^{-1}$ and $D = (0.89 \pm 0.17)\,\mu\mathrm{m}^2\,\mathrm{s}^{-1}$ for the immobile and mobile fraction in a synapse with 5c.c7 T-cells, respectively, and $D = (0.71 \pm 0.10)\,\mu\mathrm{m}^2\,\mathrm{s}^{-1}$ for the mobile fraction in a synapse with Jurkat T-cells. 724 events in 29 tracks from 109 movies for 5c.c7 cells, 561 events in 20 tracks from 68 movies for Jurkat cells.

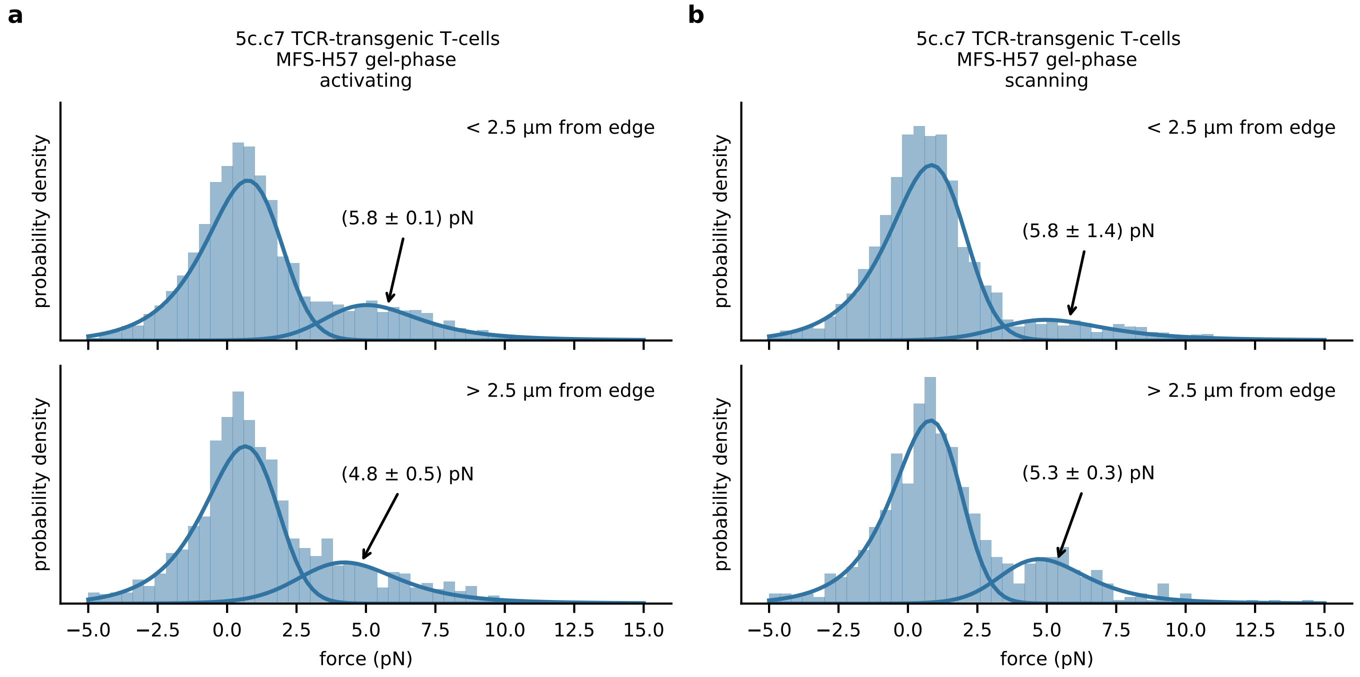

Supplementary Figure 9: *Spatial analysis of single-molecule force histograms.* We used MFS-H57 data recorded on 5c.c7 T-cells contacting activating (a) or scanning-condition (b) gel-phase SLBs. Data were grouped with respect to the closest distance of the FRET event from the cell border, representing the periphery (0 µm to 2.5 µm) or the center of the T-cell (>2.5 µm). The Gaussian mixture model revealed the indicated average forces for the high force peak. Activating conditions: <2.5 µm from edge, n = 4650 (105 movies); >2.5 µm from edge, n = 1498 (50 movies). Scanning conditions: <2.5 µm from edge, n = 3367 (92 movies); >2.5 µm from edge, n = 690 (21 movies).

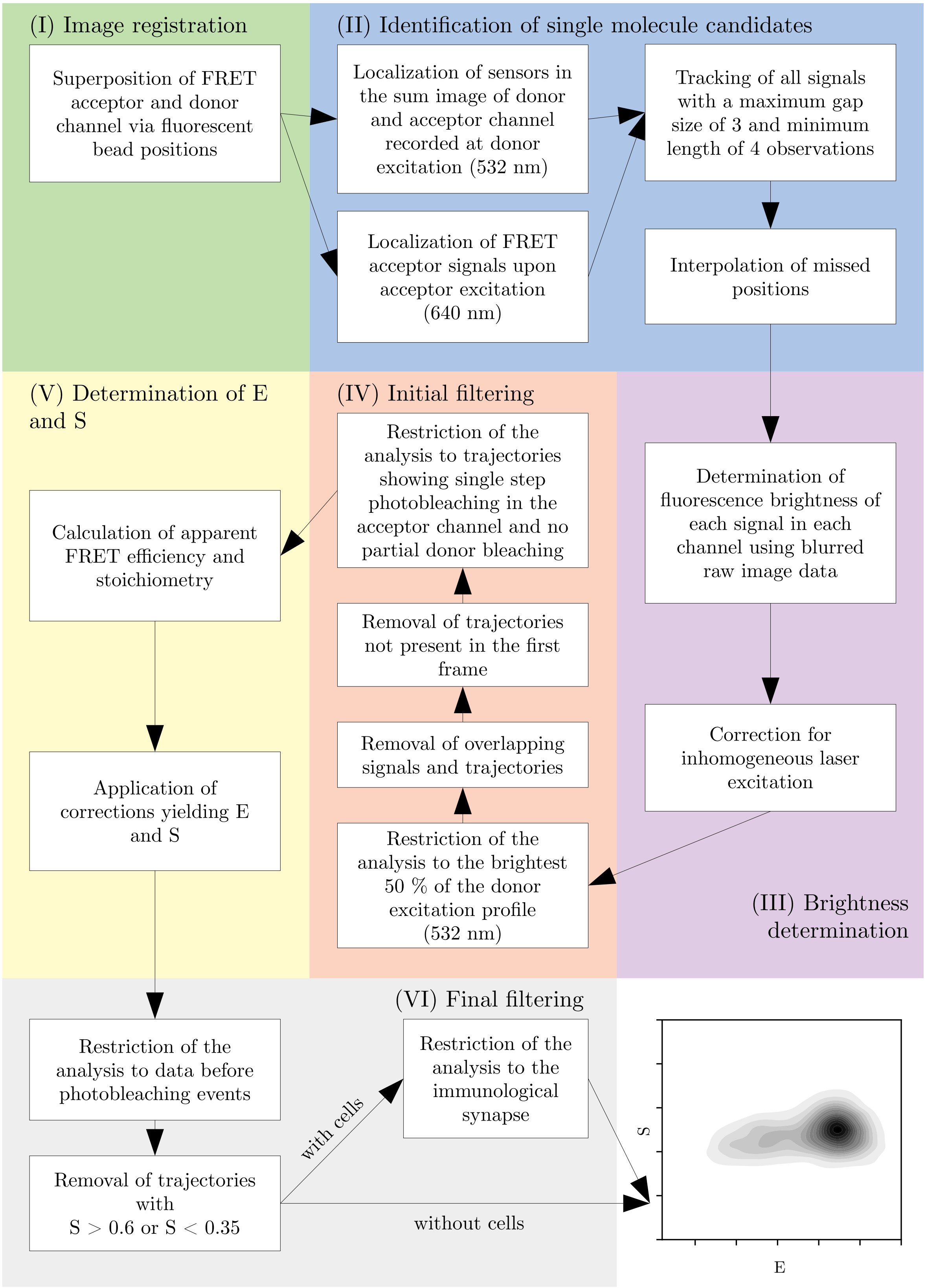

Supplementary Figure 10: *FRET analysis workflow.* Flow chart describing the single-molecule FRET analysis workflow. Subheadings relate to the description in the *Methods* section.

		low-FRET component			high-FRET component		
condition		mean	std.	weight	mean	std.	weight
5c.c7 MFS-H57 gel-phase activating	inside synapse	0.42	0.19	0.21	0.87	0.13	0.79
	no T-cells	0.89	0.29	0.09	0.86	0.11	0.91
OT-1 MFS-H57 gel-phase activating	inside synapse	0.31	0.16	0.18	0.86	0.13	0.82
	no T-cells	0.82	0.30	0.10	0.86	0.10	0.90
5c.c7 MFS-H57 gel-phase activating, DMSO	inside synapse	0.43	0.19	0.24	0.87	0.12	0.76
	no T-cells	0.78	0.36	0.04	0.87	0.10	0.96
5c.c7 MFS-H57 gel-phase activating, cytochalasin D	inside synapse	0.73	0.20	0.16	0.87	0.11	0.84
	no T-cells	0.86	0.10	0.82	0.91	0.18	0.18
5c.c7 MFS-H57 gel-phase scanning	inside synapse	0.32	0.17	0.15	0.86	0.13	0.85
	no T-cells	0.74	0.31	0.06	0.87	0.10	0.94
OT-1 MFS-H57 gel-phase scanning	inside synapse	0.39	0.14	0.15	0.86	0.14	0.85
	no T-cells	0.79	0.34	0.08	0.88	0.11	0.92
5c.c7 MFS-IE^k/MCC gel-phase activating	inside synapse	0.73	0.30	0.24	0.87	0.11	0.76
	no T-cells	0.86	0.11	0.90	0.94	0.27	0.10
5c.c7 MFS-IE^k/MCC gel-phase scanning	inside synapse	0.84	0.12	0.77	0.90	0.23	0.23
	no T-cells	0.85	0.11	0.82	0.98	0.19	0.18
5c.c7 MFS-H57 fluid-phase activating	inside synapse	0.79	0.30	0.10	0.86	0.12	0.90
	no T-cells	0.85	0.10	0.75	0.92	0.21	0.25
5c.c7 MFS-H57 fluid-phase scanning	inside synapse	0.73	0.26	0.25	0.85	0.12	0.75
	no T-cells	0.86	0.09	0.75	0.90	0.19	0.25

Supplementary Table 1: The results of a fit with a two-component Gaussian mixture model yielded the results shown in the table.
