# Supplementary material for: Temporal analysis of T-cell receptor-imposed forces via quantitative single molecule FRET measurements
Source: Nat Commun. 2021 May 4;12:2502. doi: 10.1038/s41467-021-22775-z (PMC8096839; doi:10.1038/s41467-021-22775-z)

UNICORN 6.3
User: Florian 4/27/2020 2:21:41 PM +02:00
Run By : Florian 4/27/2020 2:00:51 PM +02:00
Result: 20160902 J4 refold S200 001.pdf

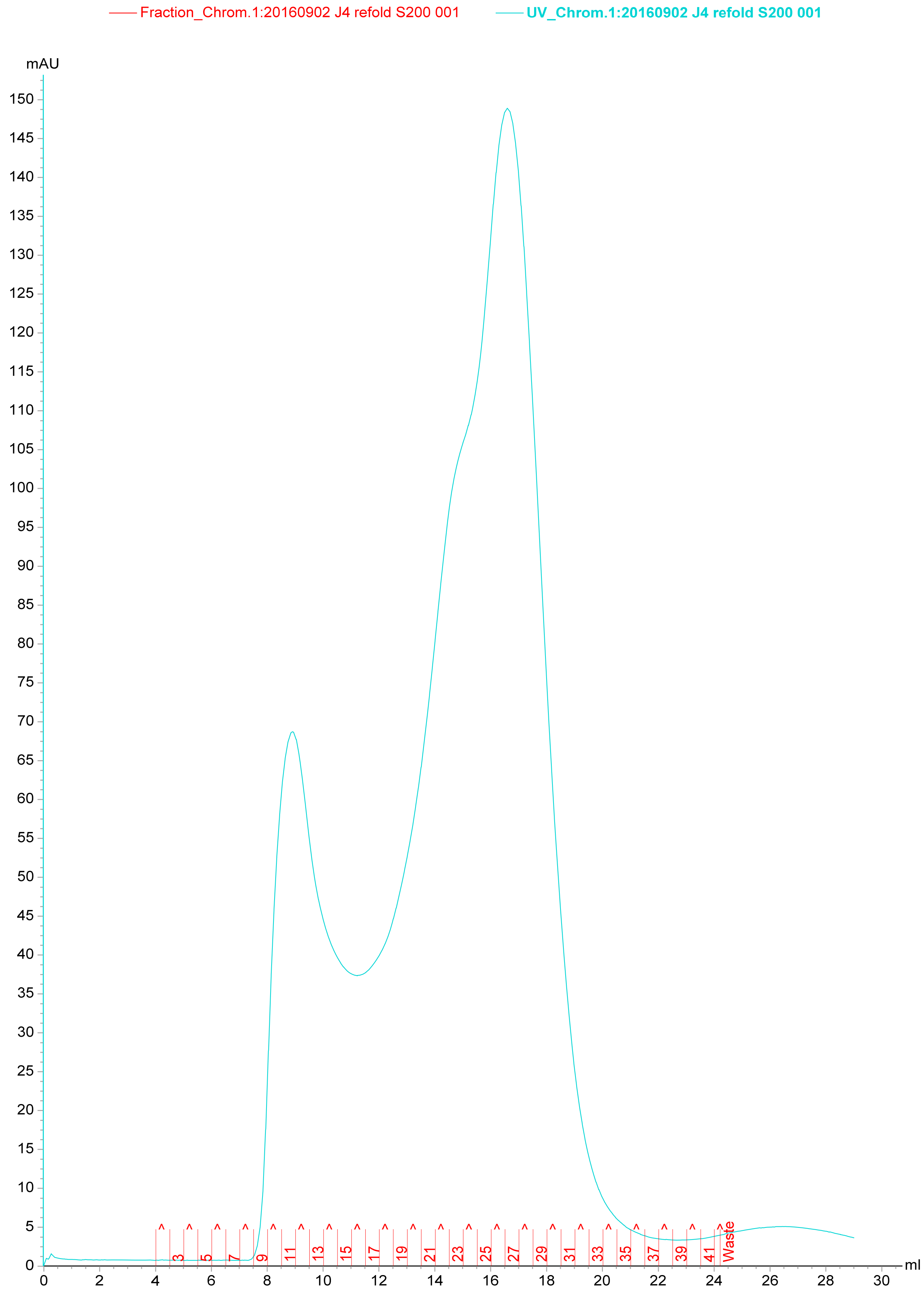

Supplement: Supplementary file 4 — Source data [file 41467_2021_22775_MOESM4_ESM.zip › Source Data/Figure_S1a/20160902_H57 cys refold S200.pdf]
