# Supplementary figures and images for: Temporal analysis of T-cell receptor-imposed forces via quantitative single molecule FRET measurements
Source: Nat Commun. 2021 May 4;12:2502. doi: 10.1038/s41467-021-22775-z (PMC8096839; doi:10.1038/s41467-021-22775-z)

User: Florian 9/9/2016 4:45:30 PM +02:00

Run By : Florian 9/9/2016 1:41:40 PM +02:00

Result: 20160909 IEk cys refold S200 001

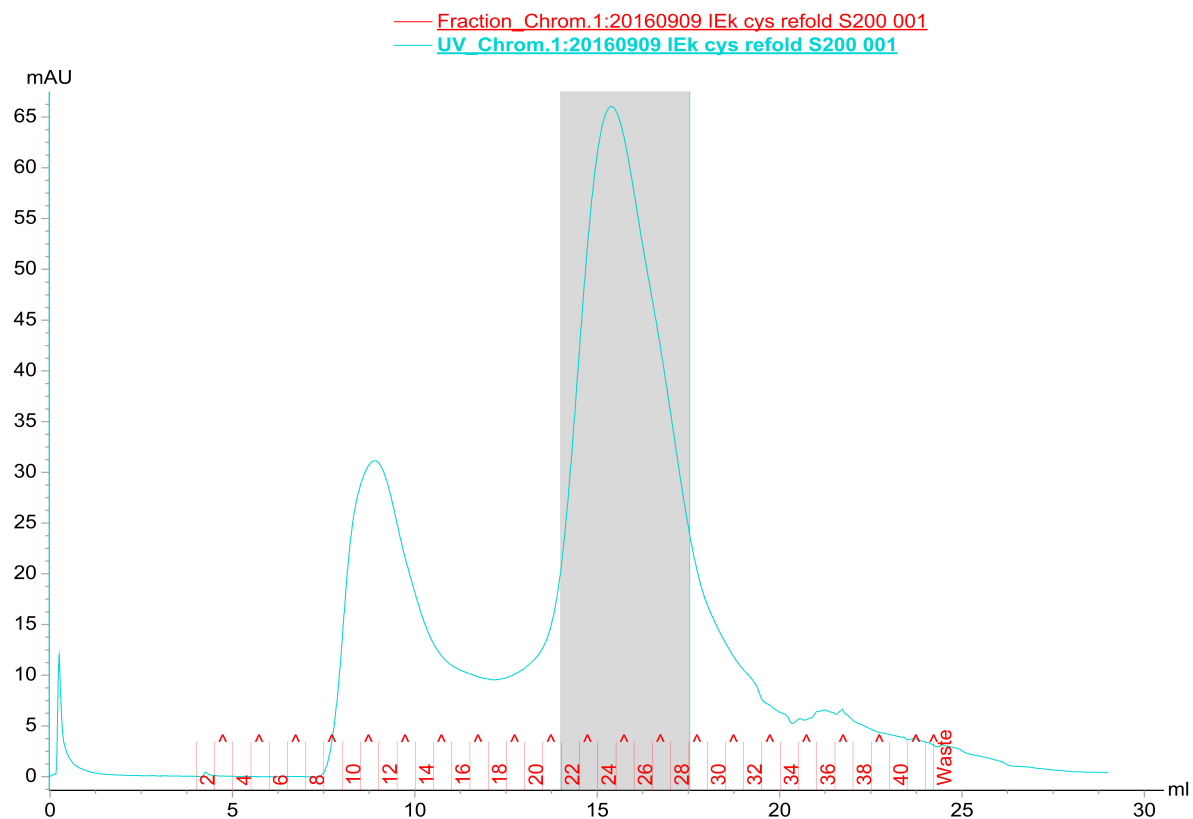

Supplement: Supplementary file 4 — Source data [file 41467_2021_22775_MOESM4_ESM.zip › Source Data/Figure_S1a/20160909 IEk cys refold S200.pdf]

UNICORN 6.3

1(1)

User: Florian 4/27/2020 2:23:36 PM +02:00

Run By : Florian 9/2/2016 2:10:12 PM +02:00

Result: 20160902 J4 DBCO S200 001

— Fraction\_Chrom.1:20160902 J4 DBCO S200 001

— UV\_Chrom.1:20160902 J4 DBCO S200 001

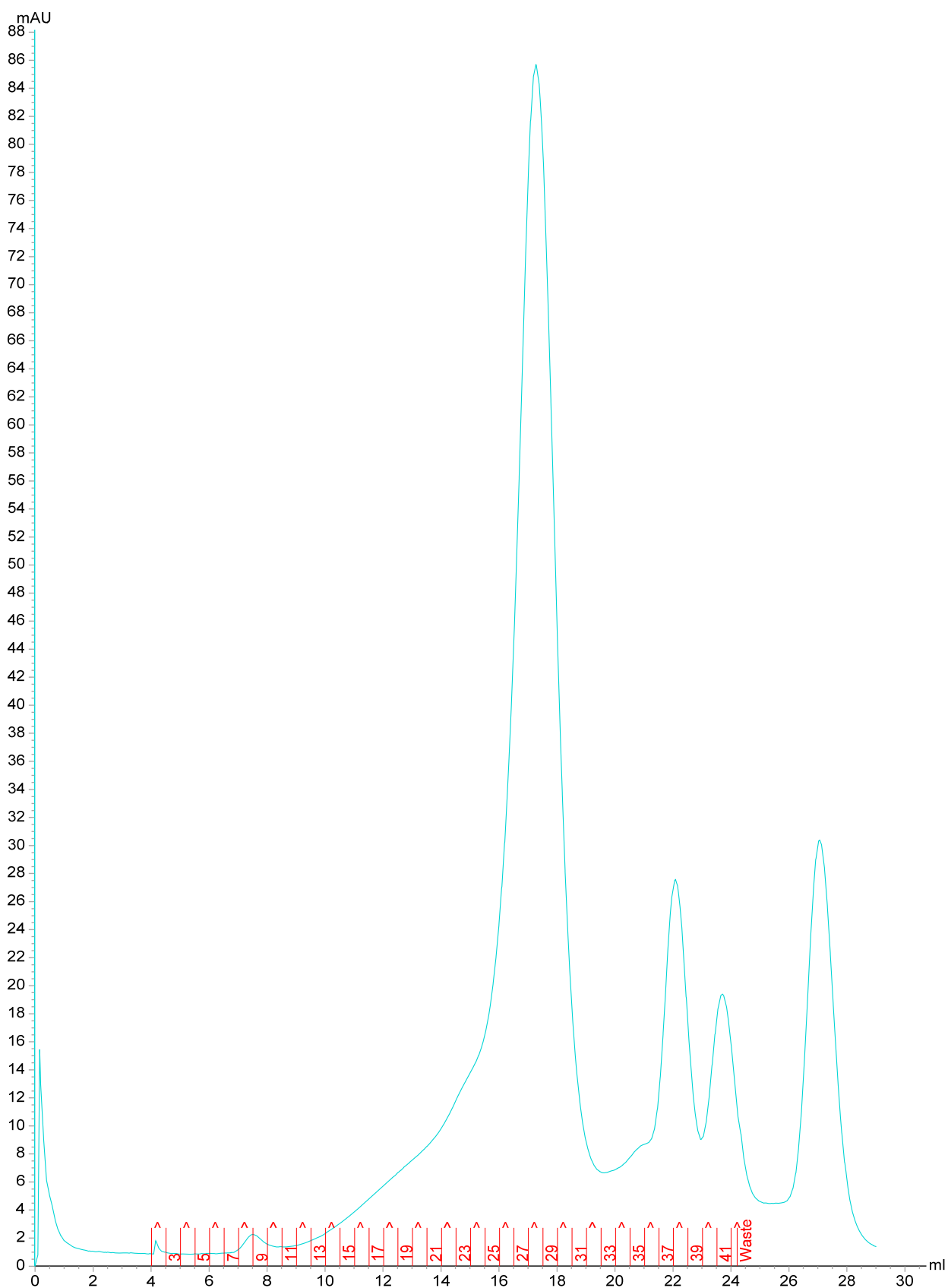

Supplement: Supplementary file 4 — Source data [file 41467_2021_22775_MOESM4_ESM.zip › Source Data/Figure_S1b/20160902_H57 DBCO S200.pdf]

User: Florian 9/9/2016 7:50:59 PM +02:00

Run By : Florian 9/9/2016 5:59:22 PM +02:00

Result: 20160909 IEk cys DBCO S200 001

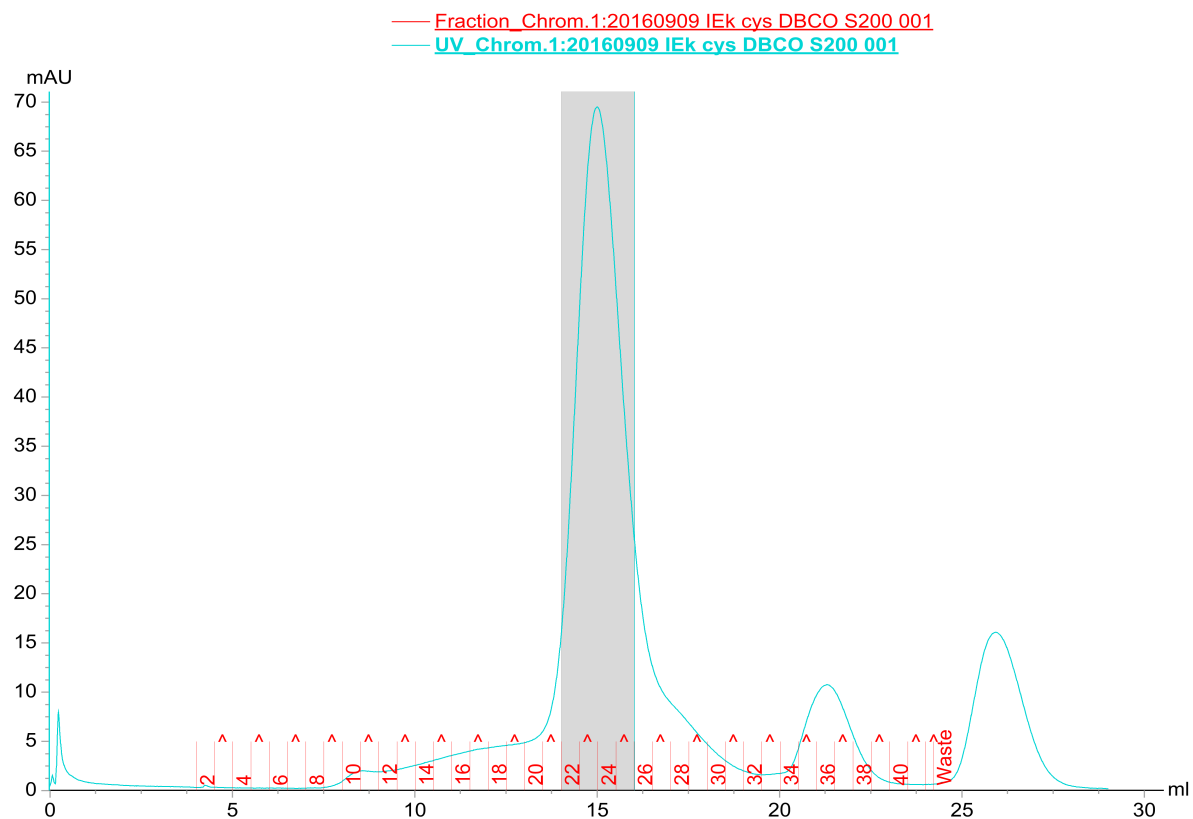

Supplement: Supplementary file 4 — Source data [file 41467_2021_22775_MOESM4_ESM.zip › Source Data/Figure_S1b/20160909 IEk DBCO S200.pdf]

User: Florian 4/27/2020 2:30:44 PM +02:00

Run By : Florian 9/22/2016 12:56:56 PM +02:00

Result: 20160922 J4 SSS 555 647 S75 001

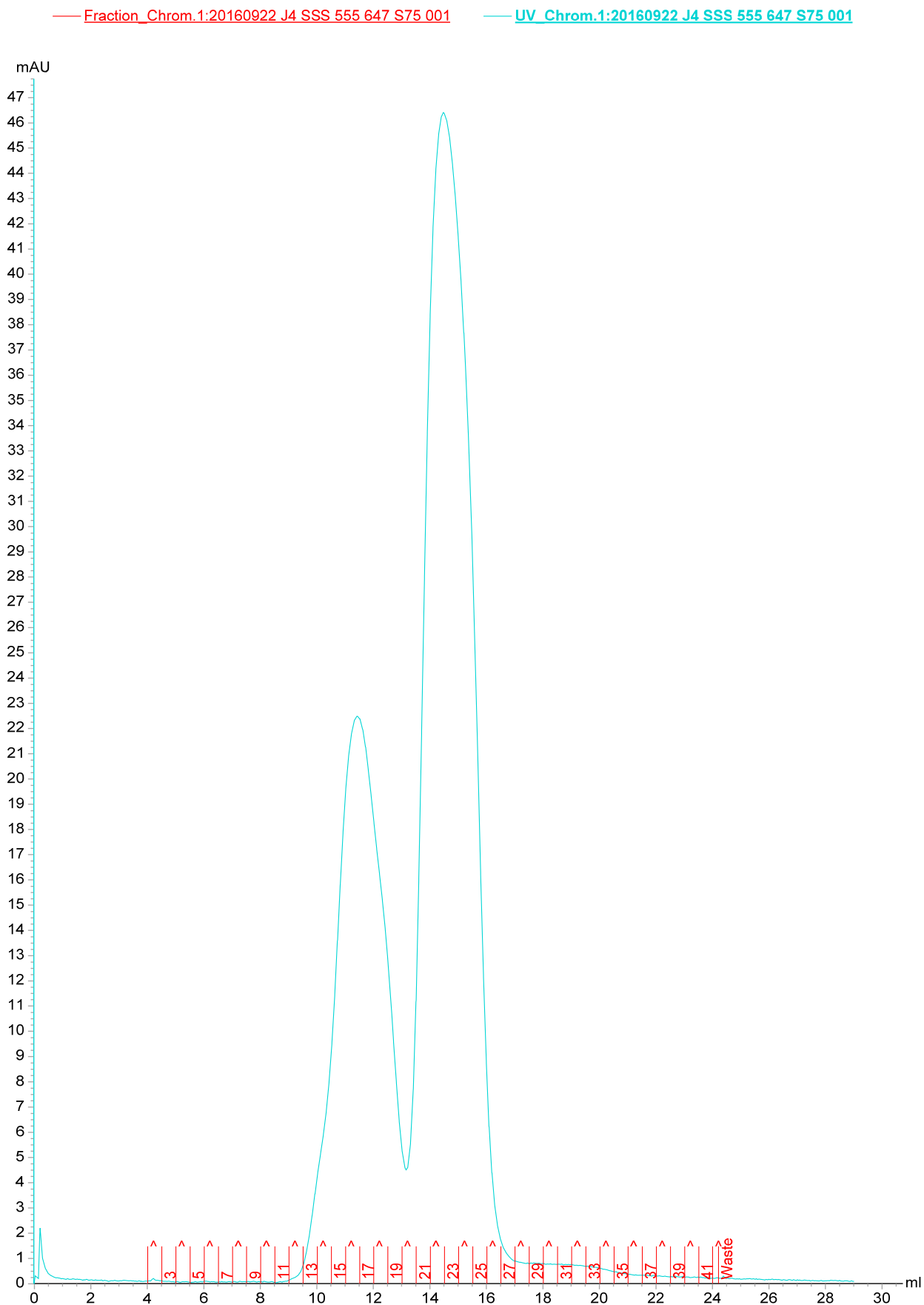

Supplement: Supplementary file 4 — Source data [file 41467_2021_22775_MOESM4_ESM.zip › Source Data/Figure_S1c/20160922_MFS-H57 S75.pdf]

User: Florian 3/8/2017 2:43:53 PM +01:00

Run By : Florian 3/8/2017 12:37:49 PM +01:00

Result: 20170308 IEk SSS5 dual S200 001

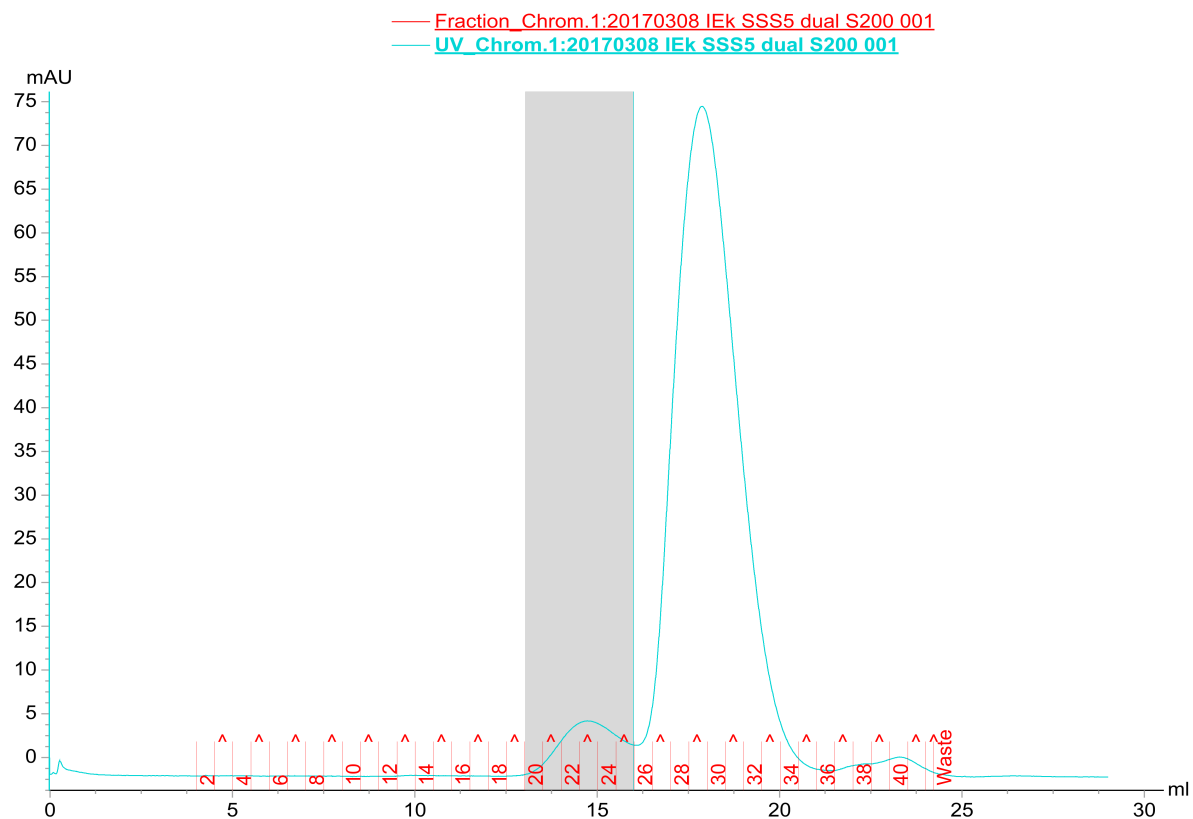

Supplement: Supplementary file 4 — Source data [file 41467_2021_22775_MOESM4_ESM.zip › Source Data/Figure_S1c/20170308_MFS-IEk-MCC S200.pdf]
